# Interhemispheric fissure distortion in fetal open spina bifida: an under‐recognized prenatal ultrasound finding

**DOI:** 10.1002/uog.70314

**Published:** 2026-08-12

**Authors:** L. S. Carmant, Y. Kunpalin, M. Kasimov, T. Van Mieghem, Y. Fisher, P. Shannon, E. Miller, S. Shinar

**Affiliations:** ^1^ Ontario Fetal Centre, Mount Sinai Hospital Toronto ON Canada; ^2^ Division of Maternal–Fetal Medicine, Department of Obstetrics & Gynaecology Mount Sinai Hospital Toronto ON Canada; ^3^ University of Toronto Toronto ON Canada; ^4^ Department of Pathology and Laboratory Medicine Mount Sinai Hospital Toronto ON Canada; ^5^ Department of Diagnostic Imaging and Interventional Radiology, Department of Medical Imaging Hospital for Sick Children Toronto ON Canada

**Keywords:** Chiari II malformation, falx cerebri, fetal surgery, interhemispheric fissure, open spina bifida, prenatal ultrasound

## Abstract

**Objectives:**

To describe, characterize and determine the frequency of interhemispheric fissure (IHF) distortion on ultrasound in fetuses with open spina bifida (OSB), evaluate its association with other intracranial findings and biometric parameters and assess its evolution after prenatal closure of the spinal defect.

**Methods:**

This retrospective cohort study included fetuses with suspected OSB referred to a single tertiary fetal center (Ontario Fetal Centre, Toronto, ON, Canada) between June 2017 and May 2025. Inclusion required prenatal diagnosis of OSB and at least one ultrasound examination with adequate axial transventricular and transthalamic planes of the fetal brain for evaluation of the IHF. IHF distortion was defined as deviation from the expected linear echogenic midline appearance. Biometric measurements were converted to gestational age‐adjusted *Z*‐scores. Associations between IHF distortion and clinical and imaging variables were assessed using univariable and multivariable logistic regression. Postoperative imaging was reviewed in fetuses that underwent prenatal spinal defect closure to evaluate changes in IHF appearance.

**Results:**

Among 133 fetuses with confirmed OSB, IHF distortion was identified in 54 (40.6%). Interobserver agreement was excellent (Cohen's kappa coefficient = 0.83). Fetuses with IHF distortion had smaller transcerebellar diameter (TCD) *Z*‐scores compared with those without distortion (−4.80 ± 2.11 *vs* −3.91 ± 2.54; *P* = 0.047). On multivariable logistic regression, later gestational age at OSB diagnosis (adjusted odds ratio (aOR), 1.43 (95% CI, 1.08–1.94); *P* = 0.01) and smaller TCD *Z*‐score (aOR, 0.89 (95% CI, 0.76–0.99); *P* = 0.04) were independently associated with IHF distortion. Among fetuses that underwent prenatal closure and had postoperative imaging available, resolution of IHF distortion paralleled improvement in hindbrain herniation, whereas persistence of IHF distortion occurred only in cases with minimal or no improvement in hindbrain herniation.

**Conclusions:**

IHF distortion is a frequent and reproducible prenatal ultrasound finding in fetuses with OSB. Its association with a smaller TCD and its apparent resolution following prenatal closure of OSB in cases that demonstrated improvement in hindbrain herniation suggest that IHF distortion may represent a secondary mechanical manifestation within the Chiari II spectrum. © 2026 The Author(s). *Ultrasound in Obstetrics & Gynecology* published by John Wiley & Sons Ltd on behalf of International Society of Ultrasound in Obstetrics and Gynecology.

## INTRODUCTION

Fetal open spina bifida (OSB) is a congenital defect resulting from failed neural tube closure in early gestation^1^. It is typically associated with a Chiari II malformation, a complex hindbrain anomaly thought to arise from chronic cerebrospinal fluid (CSF) leakage through the spinal defect. The resulting downward displacement of the cerebellum and brainstem often leads to obstructive ventriculomegaly and hydrocephalus^2^, ^3^.

In addition to classic intracranial sonographic signs of OSB, such as the ‘lemon’ and ‘banana’ signs and ventriculomegaly, a broader range of associated intracranial anomalies has been described on prenatal imaging^4^. These include posterior colpocephaly, callosal abnormalities and disorders of neuronal migration and cortical organization, including periventricular nodular heterotopias, identified on prenatal ultrasound and/or fetal magnetic resonance imaging (MRI)^5^, ^6^, ^7^, ^8^, ^9^. These findings are generally understood to fall within the broader neuroanatomical spectrum of Chiari II malformation and, as a result, do not necessarily preclude consideration for *in‐utero* closure of the defect^5^, ^7^, ^9^. More recently, our group has reported the presence of a midline suprapineal pseudocyst in fetuses with OSB^10^. We also recently identified an additional recurrent imaging feature in fetuses with OSB: distortion of the posterior aspect of the interhemispheric fissure (IHF), which is normally visualized as a thin, straight hyperechogenic line separating the cerebral hemispheres on axial and coronal views^11^, ^12^. However, in a subset of cases, the IHF appears undulating or distorted. Outside the context of OSB, distortion of the IHF is considered abnormal and has been reported in association with tubulinopathies, Aicardi syndrome, disorders of cortical development and other complex cerebral malformations^13^, ^14^, ^15^.

The primary objectives of this study were to characterize the ultrasound appearance and determine the frequency of IHF distortion in fetuses with OSB, and to evaluate its association with other intracranial findings and biometric parameters. The secondary objective was to assess the evolution of IHF distortion after fetal OSB closure.

## METHODS

### Study design

This was a retrospective cohort study conducted at the Ontario Fetal Centre (Toronto, ON, Canada), a collaboration between Mount Sinai Hospital and The Hospital for Sick Children. Data were collected between June 2017 and May 2025 from patients referred to our center during this period. The study was conducted ethically in accordance with the World Medical Association Declaration of Helsinki and reviewed and approved by the Mount Sinai Hospital and the Hospital for Sick Children Research Ethics Board (REB# 17‐0087‐C and REB# 1000080667, respectively). Written informed consent was obtained from all participants.

All fetuses referred for suspected OSB during the study period were screened for eligibility. Inclusion required prenatal OSB diagnosis and at least one ultrasound examination demonstrating adequate axial transventricular and transthalamic planes of the fetal brain, as defined by International Society of Ultrasound in Obstetrics and Gynecology (ISUOG) guidelines^11^, with satisfactory visualization of the IHF. When available, the presence or absence of distortion was corroborated on coronal views^12^. Fetuses with closed spinal defects, limited dorsal myeloschisis, chromosomal abnormalities or pathogenic copy number variants were excluded. All included cases had confirmation of OSB on postnatal MRI or postmortem examination.

Maternal, fetal and imaging data were obtained from medical records and the fetal and neonatal imaging archives. Maternal characteristics recorded included age and parity. For each fetus, gestational age (GA) at presentation, fetal sex, type of spinal lesion (classified as myeloschisis (flat lesion without a meningeal sac) or myelomeningocele (lesion with a meningeal sac)) and upper level of the lesion were recorded^16^. The level of the OSB lesion was determined using three‐dimensional (3D) ultrasound imaging by counting down from T12 to the lesion in the coronal plane of the spine or by counting up to the lesion from S4 (the lowest ossified vertebra at < 24 weeks' gestation) or S5 (the lowest ossified vertebra at ≥ 24 weeks) in the midsagittal plane^17^. Lesions were classified according to spinal level as follows: low (S2 and below), intermediate (L4–S1) and high (L3 and above)^10^. Information on prenatal management was also collected, including whether the pregnancy was managed expectantly, underwent fetal surgery or resulted in termination of pregnancy (TOP).

Ultrasound biometry collected included biparietal diameter (BPD)^18^, head circumference (HC)^19^, transcerebellar diameter (TCD)^20^ and ventricular posterior diameter (VPD)^21^. Additional intracranial findings associated with OSB were also noted, including the presence of the lemon sign, banana‐shaped cerebellum and suprapineal pseudocyst. VPD was measured in accordance with ISUOG guidelines^11^, and ventriculomegaly was classified as mild (10.0–11.9 mm), moderate (12.0–14.9 mm) or severe (≥ 15.0 mm). Biometric Z‐scores were calculated relative to GA reference charts to standardize measurements across the cohort^18^, ^19^, ^20^, ^21^.

### Ultrasound evaluation

All ultrasound examinations were performed and/or supervised by a fetal medicine specialist using either a Philips IU22 (Philips Healthcare, Amsterdam, The Netherlands) or GE Voluson E10 or Expert 22 (GE Healthcare, Zipf, Austria) machine. Most examinations were performed transabdominally as part of routine clinical fetal ultrasound assessment. Prior to 2022, dedicated fetal neurosonography according to ISUOG guidelines^12^ was not routinely performed; consequently, only 64/133 (48.1%) cases underwent dedicated neurosonographic assessment. Image review for this study was based on stored two‐dimensional ultrasound images; 3D ultrasound volumes and reconstructions of the brain were not routinely available for retrospective analysis. Digitally stored images were retrospectively and independently reviewed by two observers (L.S.C. (2 years of experience), S.S. (8 years of experience)). Both reviewers were blinded to the clinical data, postnatal findings and fetal surgical status during image review. When multiple eligible scans were available for a fetus, the first comprehensive examination with adequate axial brain views was included in the analysis. Multiple stored still images and cine clips were reviewed when available. Images with artifacts due to fetal motion or oblique scanning planes were excluded. Distortion due to artifacts was considered present only when both reviewers agreed on this finding.

**Figure 1:**
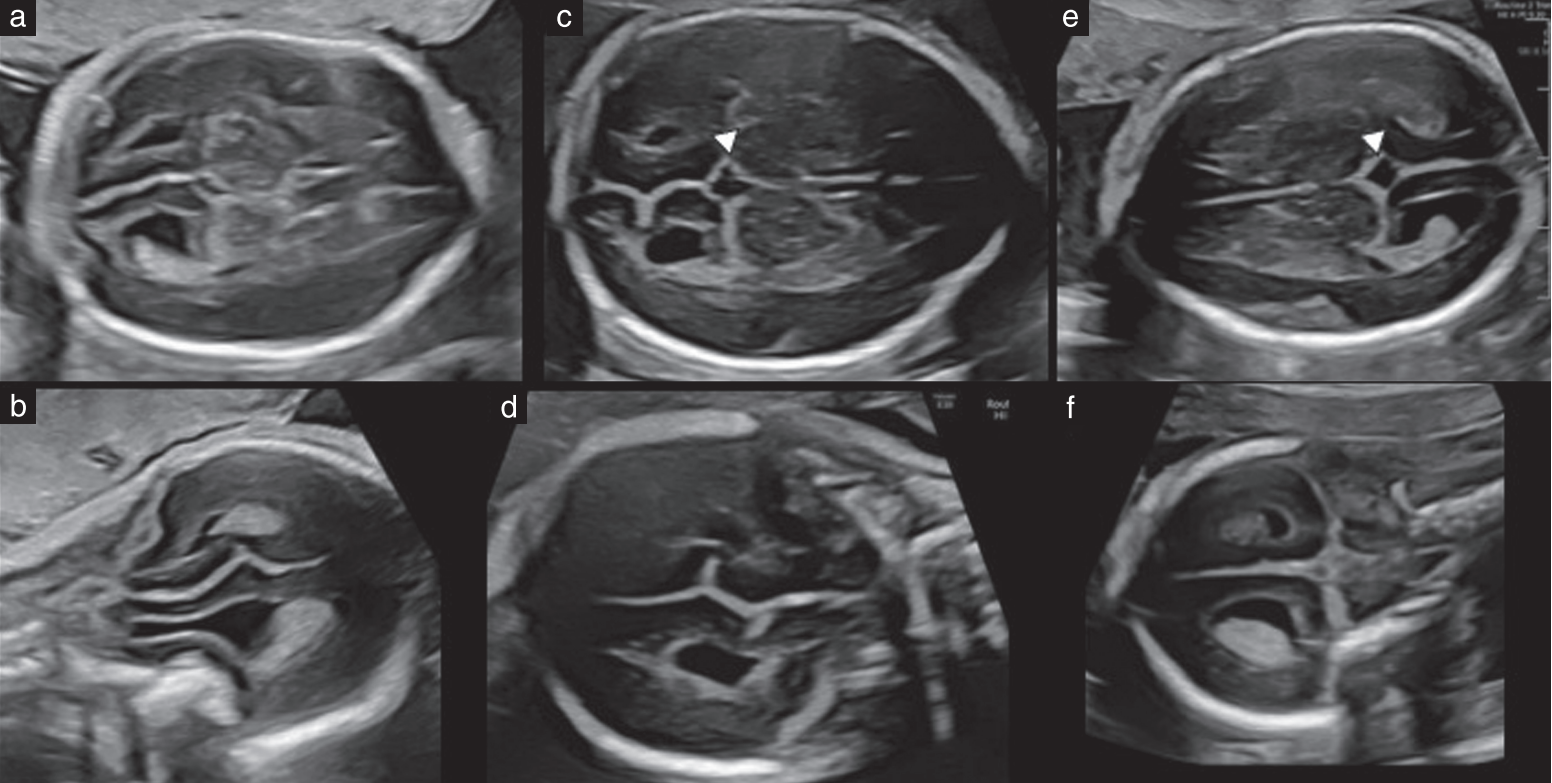
Transabdominal ultrasound images of: (a,b) fetus at 24 + 0 weeks' gestation demonstrating distortion of posterior interhemispheric fissure (IHF) in axial (a) and coronal (b) views; (c,d) fetus at 25 + 0 weeks' gestation demonstrating posterior IHF distortion in axial (c) and coronal (d) views; and (e,f) fetus at 21 + 3 weeks' gestation showing a normal posterior IHF in axial (e) and coronal (f) views. Arrowheads in (c) and (e) indicate a suprapineal pseudocyst.

The appearance of the IHF was evaluated specifically in the axial plane, as not all cases underwent dedicated neuroimaging as per ISUOG guidelines^12^. When coronal plane imaging was available, distortion was also confirmed in this plane. The IHF was considered distorted if it deviated from its expected appearance as a linear echogenic midline structure separating the cerebral hemispheres. Distortion was defined subjectively as any visible waviness, undulation or irregular contour of the posterior aspect of the IHF (Figure 1). Following fetal closure of the spinal defect, postoperative ultrasound images were analyzed to assess the impact of the surgery on the cerebellar tonsils and on the appearance of the posterior aspect of the IHF.

### MRI acquisition

Prenatal MRI was performed as part of routine clinical care, using a 3.0‐Tesla MAGNETOM Skyra scanner (Siemens Healthcare, Erlangen, Germany), according to an established institutional fetal protocol. Imaging reviewed for the purpose of this study included a single‐shot fast spin‐echo T2‐weighted sequence obtained in three orthogonal planes. The slice thickness was 3–4 mm. To assess the IHF, images were reviewed by a senior pediatric neuroradiologist (E.M. (20 years of experience)) who was blinded to the ultrasound findings.

### Postmortem neuropathological examination

Postmortem neuropathological examination was available in a subset of cases following TOP (*n* = 12). Tissue sections were reviewed independently by two neuropathologists (Y.F., P.S. (28 and 5 years of experience in perinatal neuropathology, respectively)) to assess the falx cerebri.

### Statistical analysis

Statistical analysis was performed using GraphPad Prism version 11.0 (GraphPad Software, San Diego, CA, USA). Categorical variables are presented as *n* (%), while continuous variables are reported as mean ± SD for normally distributed data and median (interquartile range (IQR)) for non‐normally distributed data. Comparisons between groups were performed using the chi‐square test or Fisher's exact test for categorical variables, and the Student's *t*‐test or Mann–Whitney *U‐*test for normally and non‐normally distributed continuous variables, respectively. Univariable and multivariable logistic regression analysis was conducted to identify factors associated with the presence of IHF distortion. Variables associated with IHF distortion at a significance level of *P* < 0.10 on univariable analysis were entered into the multivariable logistic regression model. Interobserver agreement for the assessment of IHF distortion on ultrasound was evaluated using Cohen's kappa coefficient. All tests were two‐sided and *P* < 0.05 was considered statistically significant.

As an exploratory analysis, excluded cases of closed spinal dysraphism and limited dorsal myeloschisis were also reviewed qualitatively to assess whether IHF distortion could be identified in the absence of CSF leakage.

## RESULTS

### Study population

Of 174 pregnancies referred for suspected OSB during the study period, 41 were excluded: 29 did not have OSB (18 with closed spina bifida, six with no neural tube defect and five with limited dorsal myeloschisis) and 12 had chromosomal or clinically significant copy number variants. The final cohort included 133 fetuses with confirmed OSB, of which 49 (36.8%) underwent TOP, 65 (48.9%) underwent prenatal surgical closure of the spinal defect and 19 (14.3%) underwent postnatal surgical repair.

### Maternal and fetal characteristics

Maternal and fetal characteristics of the study population are summarized in Table 1. The mean maternal age was 31.5 ± 5.7 years and 54 (40.6%) women were primiparous. Most (*n* = 104 (78.2%)) lesions were myelomeningoceles and were located at an intermediate (*n* = 78/132 (59.1%)) or high (*n* = 48/132 (36.4%)) spinal level. The median GA at first ultrasound was 22.1 (IQR, 21.1–23.4) weeks. Abnormal cranial biometry was frequent, with TCD < 5^th^ percentile in 103/121 (85.1%) cases, and BPD and HC < 5^th^ percentile in 71 (53.4%) and 62 (46.6%) cases, respectively. Ventriculomegaly was identified in 78 (58.6%) cases and was classified as severe (≥ 15 mm) in 13 (9.8%). The banana sign and lemon sign were each present in > 90% of cases (126 (94.7%) and 124 (93.2%), respectively) and a suprapineal pseudocyst was noted in 87 (65.4%) cases.

**Table 1:** Baseline maternal and fetal characteristics of pregnancies with confirmed open spina bifida (OSB) (*n* = 133)

Characteristic	Value
Maternal age (years)	31.5 ± 5.7
Primiparous	54 (40.6)
Family history of OSB	
None	125 (94.0)
First‐degree relative	1 (0.8)
Other than first‐degree relative	7 (5.3)
GA at first ultrasound (weeks)	22.1 (21.1–23.4)
Male sex	64/130 (49.2)
Type of lesion	
Myeloschisis	29 (21.8)
Myelomeningocele	104 (78.2)
Upper level of lesion	
Low (≤ S2)	6/132 (4.5)
Intermediate (L4–S1)	78/132 (59.1)
High (≥ L3)	48/132 (36.4)
Biparietal diameter at first ultrasound*	
< 5^th^ percentile	71 (53.4)
5^th^–95^th^ percentile	61 (45.9)
> 95^th^ percentile	1 (0.8)
Head circumference at first ultrasound*	
< 5^th^ percentile	62 (46.6)
5^th^–95^th^ percentile	70 (52.6)
> 95^th^ percentile	1 (0.8)
Transcerebellar diameter at first ultrasound*, †	
< 5^th^ percentile	103/121 (85.1)
5^th^–95^th^ percentile	18/121 (14.9)
> 95^th^ percentile	0/121 (0)
Ventriculomegaly at first ultrasound*, ‡	
None	55 (41.4)
Mild	34 (25.6)
Moderate	31 (23.3)
Severe	13 (9.8)
Other neuroimaging findings	
Banana sign	126 (94.7)
Lemon sign	124 (93.2)
Suprapineal pseudocyst	87 (65.4)

Data are presented as mean ± SD, *n* (%), median (interquartile range) or *n*/*N* (%).

* Biparietal diameter, head circumference, transcerebellar diameter and ventricular posterior diameter (VPD) measurements were interpreted using previously published reference charts^18–21^.

† In 12 fetuses, the cerebellum was not measurable owing to significant herniation.

‡ Classified according to VPD as mild (10.0–11.9 mm), moderate (12.0–14.9 mm) or severe (≥ 15.0 mm). GA, gestational age.

### Interhemispheric fissure distortion

Distortion of the IHF was identified on prenatal ultrasound in 54 (40.6%) fetuses. Interobserver agreement for IHF distortion on ultrasound was excellent, with a Cohen's kappa coefficient of 0.83 (*P* < 0.0001). Review of the corresponding prenatal MRI demonstrated that supratentorial crowding of the cerebral hemispheres resulted in poor spatial resolution, limiting reliable assessment of the IHF and precluding reliable correlation between ultrasound and MRI findings in this study.

During neuropathological examination, assessment of IHF and falcine morphology was limited by postmortem artifacts, including transection of the falx during extraction, maceration, deformation during handling and settling during fixation. Agonal hemorrhage further obscured subtle *in vivo* anatomical relationships in some cases. As a result, pathological correlation with prenatal imaging was limited. Furthermore, not all cases had corresponding prenatal coronal neurosonographic images available, precluding direct correlation.

Comparison of parameters in fetuses with and without IHF distortion is shown in Table 2. Fetuses with IHF distortion had significantly smaller TCD *Z*‐scores (−4.80 ± 2.11 *vs* −3.91 ± 2.54; *P* = 0.047), but no significant differences in GA at OSB diagnosis, lesion type, lesion level, or BPD, HC or VPD Z‐scores were observed. Suprapineal pseudocysts were more frequent among fetuses with IHF distortion (74.1% *vs* 59.5%), although the difference did not reach statistical significance (*P* = 0.08). The prevalence of the banana sign and that of the lemon sign were comparable (*P* = 0.70 and *P* = 0.48, respectively).

**Table 2:** Characteristics of fetuses with open spina bifida (OSB) (*n* = 133), according to whether interhemispheric fissure (IHF) distortion was present or absent

Parameter	IHF distortion present (*n* = 54)	IHF distortion absent (*n* = 79)	*P*
GA at OSB diagnosis (weeks)	22.4 (21.3–23.7)	22.0 (21.0–23.0)	0.07
Type of OSB			0.07
Myeloschisis	16 (29.6)	13 (16.5)	
Myelomeningocele	38 (70.4)	66 (83.5)	
Lesion level*			0.64
Low	2/53 (3.8)	4 (5.1)	
Intermediate	34/53 (64.2)	44 (55.7)	
High	17/53 (32.1)	31 (39.2)	
BPD *Z*‐score†	−1.15 (−2.00 to −0.15)	−1.04 (−1.79 to −0.41)	0.64
HC *Z*‐score†	−0.98 (−1.55 to −0.71)	−1.12 (−1.42 to −0.49)	0.50
TCD *Z*‐score†	−4.80 ± 2.11	−3.91 ± 2.54	0.047
VPD *Z*‐score†	3.42 ± 3.83	3.58 ± 3.16	0.79
Other neuroimaging findings			
Banana sign	52 (96.3)	74 (93.7)	0.70
Lemon sign	49 (90.7)	75 (94.9)	0.48
Suprapineal pseudocyst	40 (74.1)	47 (59.5)	0.08

Data are presented as median (interquartile range), *n* (%), *n*/*N* (%) or mean ± SD.

* The upper level of the lesion, determined using three‐dimensional ultrasound, was considered the lesion level and classified as low (S2 and below), intermediate (L4–S1) and high (L3 and above)^10^.

† *Z*‐scores for biparietal diameter (BPD), head circumference (HC), transcerebellar diameter (TCD) and ventricular posterior diameter (VPD) were calculated relative to gestational age (GA) reference charts^18–21^. *Z*‐scores for TCD and VPD were normally distributed, hence are given as mean ± SD.

### Factors associated with interhemispheric fissure distortion

On univariable logistic regression (Table 3), a smaller TCD Z‐score was significantly associated with the presence of IHF distortion (odds ratio (OR), 0.85 (95% CI, 0.72–0.99); *P* = 0.04). Later GA at diagnosis and the presence of a suprapineal pseudocyst showed a trend toward an association with IHF distortion (OR, 1.23 (95% CI, 0.99–1.54), *P* = 0.06 and OR, 1.95 (95% CI, 0.93–4.23), *P* = 0.08, respectively), but statistical significance was not reached. There was also a trend towards a lower likelihood of IHF distortion in myelomeningocele compared with myeloschisis (OR, 0.47 (95% CI, 0.20–1.08); *P* = 0.07), although this also did not reach statistical significance. No significant associations were observed for lesion level or head biometry.

**Table 3:** Univariable and multivariable logistic regression analyses for factors associated with interhemispheric fissure (IHF) distortion in fetuses with open spina bifida (OSB)

Parameter	Unadjusted OR (95% CI)	*P*	Adjusted OR (95% CI)*	*P*
GA at OSB diagnosis (weeks)	1.23 (0.99–1.54)	0.06	1.43 (1.08–1.94)	0.01
Myelomeningocele†	0.47 (0.20–1.08)	0.07	1.98 (0.78–5.10)	0.15
Lesion level	0.83 (0.44–1.55)	0.55	—	—
BPD *Z*‐score‡	0.90 (0.68–1.12)	0.46	—	—
HC *Z*‐score‡	0.90 (0.58–1.03)	0.16	—	—
TCD *Z*‐score‡	0.85 (0.72–0.99)	0.04	0.89 (0.76–0.99)	0.04
VPD *Z*‐score‡	0.95 (0.86–1.06)	0.38	—	—
Suprapineal pseudocyst	1.95 (0.93–4.23)	0.08	—	—

* Variables with *P* < 0.10 on univariable analysis were entered into the multivariable model.

† Compared with cases with myeloschisis as the reference standard.

‡ *Z*‐scores for biparietal diameter (BPD), head circumference (HC), transcerebellar diameter (TCD) and ventricular posterior diameter (VPD) were calculated relative to gestational age (GA) reference charts^18–21^. OR, odds ratio.

On multivariable logistic regression (Table 3), later GA at OSB diagnosis (adjusted OR, 1.43 (95% CI, 1.08–1.94); *P* = 0.01) and smaller TCD *Z*‐score (adjusted OR, 0.89 (95% CI, 0.76–0.99); *P* = 0.04) remained independently associated with the presence of IHF distortion.

### Impact of prenatal closure of open spina bifida on interhemispheric fissure distortion

**Figure 2:**
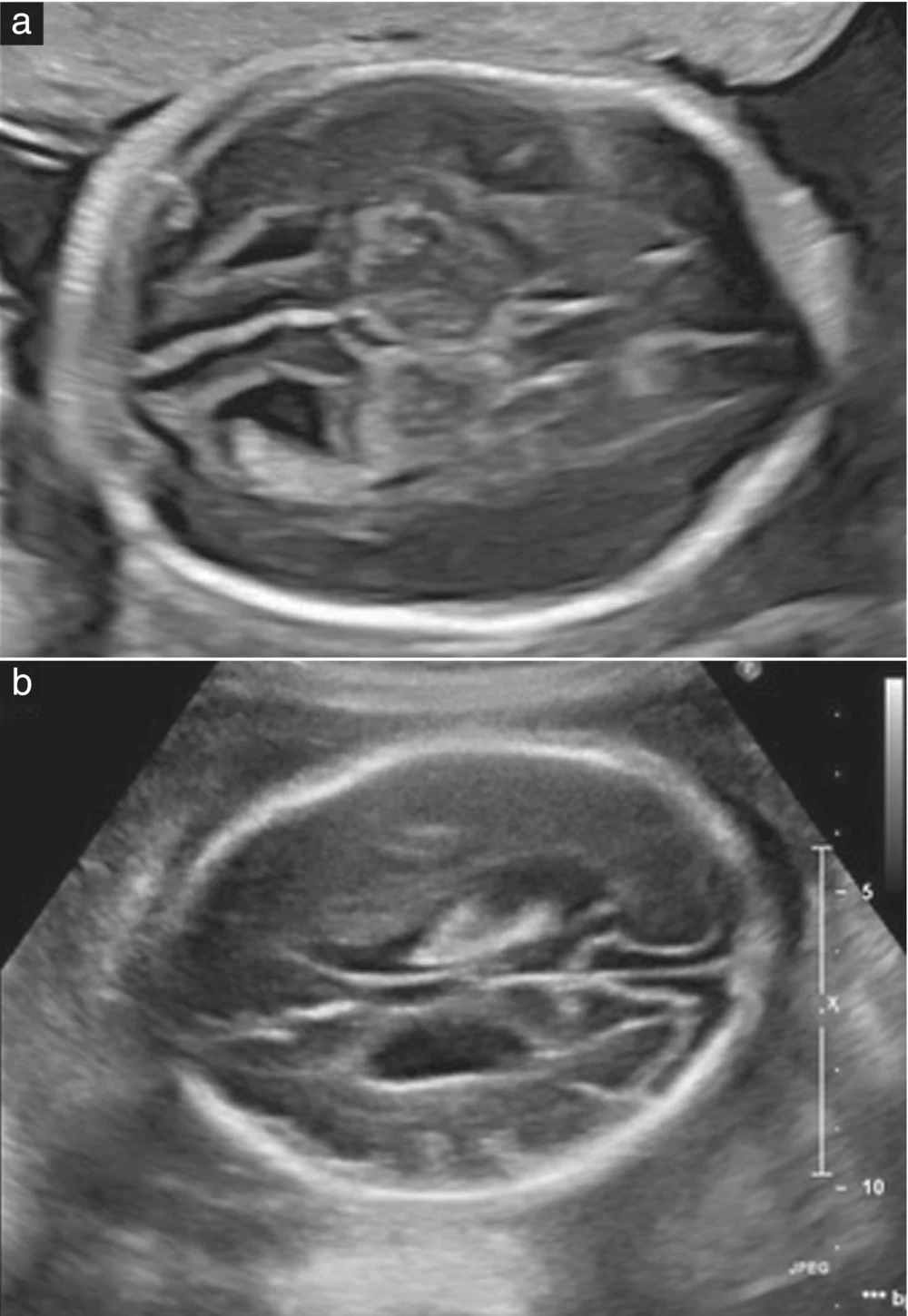
Transabdominal ultrasound imaging of fetus at 24 + 0 weeks' gestation in axial view, demonstrating distortion of the posterior interhemispheric fissure (IHF) prior to fetal myelomeningocele repair (a) and the same fetus at 31 + 1 weeks' gestation in axial view, demonstrating a normal‐appearing IHF following fetal myelomeningocele repair (b).

Among 65 fetuses that underwent prenatal closure of the spinal defect, 28 (43.1%) demonstrated IHF distortion at the first ultrasound scan before surgery. Thirteen of these cases had postoperative imaging available at a median of 6 (IQR, 5–7) weeks after closure, when improvement in hindbrain herniation is typically expected^22^. Of these, four (30.8%) continued to show IHF distortion, all of which also demonstrated little or no qualitative improvement in hindbrain herniation on the sagittal plane. In contrast, the remaining nine (69.2%) showed complete resolution of IHF distortion in parallel with marked improvement in hindbrain herniation (Figure 2). Among the 37 fetuses without IHF distortion before prenatal closure, 19 had postoperative imaging; none of these cases developed new IHF distortion and all demonstrated qualitative improvement in hindbrain herniation.

### Exploratory analysis of excluded spinal dysraphism cases

As an exploratory analysis, the morphology of the IHF was reviewed in excluded cases of closed spinal dysraphism (*n* = 18) and limited dorsal myeloschisis (*n* = 5). In cases of closed spinal dysraphism, the IHF consistently appeared straight. In contrast, among cases of limited dorsal myeloschisis, IHF distortion was observed in two cases with postnatal evidence of CSF leakage.

## DISCUSSION

### Principal findings

In this study we describe a previously unreported finding identified on prenatal ultrasound in fetuses with OSB: distortion of the IHF, which was identified in approximately 40% of cases and demonstrated excellent interobserver agreement. It was associated with smaller TCD and later GA at OSB diagnosis and, in a subset of cases, appeared to improve following prenatal closure of the spinal defect, in parallel with qualitative improvement in hindbrain herniation. Together, these findings suggest that IHF distortion represents a relatively common sonographic feature within the spectrum of intracranial changes observed in OSB.

### Interpretation of findings

The association between IHF distortion and a smaller TCD supports a relationship with posterior fossa crowding, a central feature of Chiari II malformation. According to the ‘unified theory’, chronic CSF leakage through the open spinal defect leads to reduced intracranial CSF volume, impaired ventricular expansion and underdevelopment of the posterior fossa, resulting in downward displacement of the hindbrain^23^. Within this framework, IHF distortion may reflect secondary deformation of the falx cerebri owing to altered intracranial pressure and supratentorial crowding.

The observed association of IHF distortion with GA at OSB diagnosis raises the possibility that IHF distortion may evolve over time, potentially reflecting progressive intracranial deformation. However, longitudinal imaging studies using both prenatal ultrasound and prenatal MRI are required to confirm whether distortion develops or becomes more pronounced with advancing gestation.

The trend toward association with suprapineal pseudocysts further supports a shared underlying mechanism related to altered CSF dynamics and midline traction.

### Evolution following prenatal closure of the spinal defect

In a subset of fetuses that underwent prenatal closure of OSB, complete resolution of IHF distortion was observed in parallel with qualitative improvement in hindbrain herniation, whereas persistence occurred only in cases with limited or absent improvement in hindbrain herniation. Although based on a small sample, these findings support a secondary, potentially reversible mechanical process. The apparent reversibility of IHF distortion after fetal closure of the spinal defect further reinforces that it is probably related to altered CSF dynamics rather than that it is a fixed structural abnormality.

While based on very small numbers (*n* = 23), the exploratory analysis further supports the hypothesis that IHF distortion may arise in the context of altered CSF dynamics and intracranial pressure relationships. The absence of distortion in closed spinal dysraphism, compared with its presence in limited dorsal myeloschisis cases with evidence of CSF leakage, is consistent with a secondary mechanical process rather than a primary structural abnormality.

### Comparison with existing literature

To our knowledge, IHF distortion has not been described previously on prenatal ultrasound in OSB. Postnatal MRI studies on Chiari II malformation have reported interhemispheric cortical interdigitations attributed to falcine hypoplasia, allowing cortical gyri to extend across the midline^24^. In contrast, the sonographic appearance described in our study appears more consistent with deformation of an otherwise intact falx cerebri in the setting of reduced CSF volume and supratentorial crowding.

Supporting this interpretation, Bruzek *et al*.^25^ reported that falx hypoplasia was significantly more common in patients undergoing postnatal closure of the spinal defect compared with those treated prenatally. This raises the possibility that falcine distortion may initially represent a dynamic and potentially reversible process, with prolonged mechanical deformation leading to structural alteration over time.

### Clinical implications

Recognition of IHF distortion may provide an additional sonographic feature in fetuses with OSB. As a relatively common and probably reversible feature, its presence should not be considered a contraindication to fetal surgery, but rather an associated anomaly.

In our cohort, the absence of IHF distortion prior to surgery was consistently associated with qualitative improvement in hindbrain herniation following prenatal OSB closure, whereas persistence of IHF distortion was observed only when improvement in hindbrain herniation was limited. Although these findings require confirmation in larger cohorts, assessment of IHF morphology on prenatal ultrasound may offer additional information during preoperative counseling regarding the likelihood of postoperative hindbrain herniation improvement. The persistence of IHF distortion on follow‐up ultrasound may also serve as a marker of incomplete reversal of Chiari II features after closure.

### Strengths and limitations

The strengths of this study include the relatively large single‐center cohort, standardized imaging within an experienced fetal center and the blinded independent review by two senior sonographers. Predefined imaging criteria and the use of the first detailed ultrasound at our center ensured consistency in image quality and interpretation.

Limitations include the retrospective design and the subjective nature of IHF distortion assessment, which carries an inherent risk of observer bias despite excellent interobserver agreement. In addition, no objective quantitative measure of falcine deviation was available. Future studies could aim to develop standardized and objective assessment methods, potentially including angular measurements of falcine deviation from the midline using standardized axial and coronal neurosonographic planes compared with normal controls. In addition, transvaginal neurosonography and 3D ultrasound reconstruction were not systematically available in this retrospective cohort, and may have improved assessment of associated cortical abnormalities had they been available. The relatively limited sample size and number of variables included in the multivariable regression analysis may also have increased the risk of model overfitting; therefore, the multivariable findings should be interpreted with caution and considered hypothesis‐generating. Assessment of postoperative evolution was limited by the relatively small number of cases with available follow‐up imaging, as many patients returned to their referring centers for follow‐up. In addition, although a subset of fetuses underwent third‐trimester imaging, systematic reassessment of IHF morphology later in gestation was not performed in expectantly managed cases, and many pregnancies underwent TOP prior to third‐trimester follow‐up. Therefore, the temporal evolution of this finding throughout gestation could not be fully assessed. Postnatal imaging and outcomes were not systematically evaluated in this study, precluding correlation with long‐term outcomes. Finally, the absence of a control group limited assessment of specificity, although this appearance is not observed in normal fetal brains.

### Conclusions

IHF distortion is a frequent and reproducible prenatal ultrasound finding in fetuses with OSB. Its association with a smaller TCD and its apparent reversibility following fetal closure of the spinal defect suggest that it reflects a secondary intracranial deformation related to altered CSF dynamics within the Chiari II spectrum. As such, recognition of this feature alone should not be considered a contraindication to fetal surgery, and its evolution following surgery warrants further study as a possible indicator of resolution of hindbrain herniation.

## Data Availability

The data that support the findings of this study are available on request from the corresponding author. The data are not publicly available due to privacy or ethical restrictions.

## References

[1] Copp AJ , Adzick NS , Chitty LS , Fletcher JM , Holmbeck GN , Shaw GM . Spina bifida. Nat Rev Dis Primers. 2015;1:15007. 27189655 10.1038/nrdp.2015.7 PMC4898641

[2] Juranek J , Salman MS . Anomalous development of brain structure and function in spina bifida myelomeningocele. Dev Disabil Res Rev. 2010;16(1):23‐30. 20419768 10.1002/ddrr.88 PMC2917986

[3] Trudell AS , Odibo AO . Diagnosis of spina bifida on ultrasound: always termination? Best Pract Res Clin Obstet Gynaecol. 2014;28(3):367‐377. 24373566 10.1016/j.bpobgyn.2013.10.006

[4] Nicolaides KH , Campbell S , Gabbe SG , Guidetti R . Ultrasound screening for spina bifida: cranial and cerebellar signs. Lancet. 1986;2(8498):72‐74. 2425202 10.1016/s0140-6736(86)91610-7

[5] Trigo L , Eixarch E , Bottura I , et al. Prevalence of supratentorial anomalies assessed by magnetic resonance imaging in fetuses with open spina bifida. Ultrasound Obstet Gynecol. 2022;59(6):804‐812. 34396624 10.1002/uog.23761

[6] Kunpalin Y , Deprest J , Papastefanou I , et al. Incidence and patterns of abnormal corpus callosum in fetuses with isolated spina bifida aperta. Prenat Diagn. 2021;41(8):957‐964. 33834531 10.1002/pd.5945 PMC7613455

[7] Kunpalin Y , Richter J , Mufti N , et al. Cranial findings detected by second‐trimester ultrasound in fetuses with myelomeningocele: a systematic review. BJOG. 2021;128(2):366‐374. 32926566 10.1111/1471-0528.16496 PMC8436766

[8] Maurice P , Garel J , Garel C , et al. New insights in cerebral findings associated with fetal myelomeningocele: a retrospective cohort study in a single tertiary centre. BJOG. 2021;128(2):376‐383. 32112473 10.1111/1471-0528.16185

[9] Mufti N , Sacco A , Aertsen M , et al. What brain abnormalities can magnetic resonance imaging detect in foetal and early neonatal spina bifida: a systematic review. Neuroradiology. 2022;64(2):233‐245. 34792623 10.1007/s00234-021-02853-1 PMC8789702

[10] Kunpalin Y , Sichitiu J , Krishan P , et al. Midline suprapineal pseudocyst in brain of fetuses with open spina bifida. Ultrasound Obstet Gynecol. 2023;62(3):383‐390. 37058393 10.1002/uog.26221

[11] Malinger G , Paladini D , Haratz KK , Monteagudo A , Pilu GL , Timor‐Tritsch IE . ISUOG Practice Guidelines (updated): sonographic examination of the fetal central nervous system. Part 1: performance of screening examination and indications for targeted neurosonography. Ultrasound Obstet Gynecol. 2020;56(3):476‐484. 32870591 10.1002/uog.22145

[12] Paladini D , Malinger G , Birnbaum R , et al. ISUOG Practice Guidelines (updated): sonographic examination of the fetal central nervous system. Part 2: performance of targeted neurosonography. Ultrasound Obstet Gynecol. Apr 2021;57(4):661‐671. 33734522 10.1002/uog.23616

[13] Cabet S , Karl K , Garel C , et al. Two different prenatal imaging cerebral patterns of tubulinopathy. Ultrasound Obstet Gynecol. 2021;57(3):493‐497. 32149430 10.1002/uog.22010

[14] Pomar L , Ochoa J , Cabet S , et al. Prenatal diagnosis of Aicardi syndrome based on a suggestive imaging pattern: a multicenter case‐series. Prenat Diagn. 2022;42(4):484‐494. 34984691 10.1002/pd.6085 PMC9302986

[15] Vinurel N , Van Nieuwenhuyse A , Cagneaux M , et al. Distortion of the anterior part of the interhemispheric fissure: significance and implications for prenatal diagnosis. Ultrasound Obstet Gynecol. 2014;43(3):346‐352. 23640781 10.1002/uog.12498

[16] Muller JM , Corral Sereno E , Jimenez A , et al. Differences between myeloschisis and myelomeningocele in patients undergoing prenatal repair of open spina bifida. Fetal Diagn Ther. 2025;52(2):114‐123. 38471477 10.1159/000538099 PMC11981588

[17] Coleman BG , Langer JE , Horii SC . The diagnostic features of spina bifida: the role of ultrasound. Fetal Diagn Ther. 2015;37(3):179‐196. 25341807 10.1159/000364806

[18] Chitty LS , Altman DG , Henderson A , Campbell S . Charts of fetal size: 2. head measurements. Br J Obstet Gynaecol. 1994;101(1):35‐43. 8297866 10.1111/j.1471-0528.1994.tb13007.x

[19] Chervenak FA , Jeanty P , Cantraine F , et al. The diagnosis of fetal microcephaly. Am J Obstet Gynecol. 1984;149(5):512‐517. 6742021 10.1016/0002-9378(84)90027-9

[20] Sherer DM , Sokolovski M , Dalloul M , Pezzullo JC , Osho JA , Abulafia O . Nomograms of the axial fetal cerebellar hemisphere circumference and area throughout gestation. Ultrasound Obstet Gynecol. 2007;29(1): 32‐37. 17171631 10.1002/uog.3879

[21] Snijders RJ , Nicolaides KH . Fetal biometry at 14‐40 weeks' gestation. Ultrasound Obstet Gynecol. 1994;4(1):34‐48. 12797224 10.1046/j.1469-0705.1994.04010034.x

[22] Mufti N , Chappell J , Aertsen M , et al. Assessment of longitudinal brain development using super‐resolution magnetic resonance imaging following fetal surgery for open spina bifida. Ultrasound Obstet Gynecol. 2023;62(5):707‐720. 37161647 10.1002/uog.26244 PMC10947002

[23] McLone DG , Knepper PA . The cause of Chiari II malformation: a unified theory. Pediatr Neurosci. 1989;15(1):1‐12. 2699756 10.1159/000120432

[24] Miller E , Widjaja E , Blaser S , Dennis M , Raybaud C . The old and the new: supratentorial MR findings in Chiari II malformation. Childs Nerv Syst. 2008;24(5):563‐575. 18026960 10.1007/s00381-007-0528-x

[25] Bruzek AK , Koller GM , Karuparti S , et al. MRI analysis of neurodevelopmental anatomy in myelomeningocele: prenatal vs postnatal repair. Ultrasound Obstet Gynecol. 2024;64(3):362‐373. 38237046 10.1002/uog.27586

